# Study protocol for a randomized controlled trial to explore the effects of personalized lifestyle advices and tandem skydives on pleasure in anhedonic young adults

**DOI:** 10.1186/s12888-016-0880-z

**Published:** 2016-06-04

**Authors:** Eeske van Roekel, Maurits Masselink, Charlotte Vrijen, Vera E. Heininga, Tom Bak, Esther Nederhof, Albertine J. Oldehinkel

**Affiliations:** University of Groningen, University Medical Center Groningen, Interdisciplinary Center Psychopathology and Emotion regulation, CC 72, P.O. Box 30001, 9700 RB Groningen, The Netherlands; Van Hall Larenstein, University of Applied Science, Leeuwarden, The Netherlands

**Keywords:** Anhedonia, Intervention, Personalized lifestyle advice, Tandem skydive, Young adults, Randomized controlled trial

## Abstract

**Background:**

Anhedonia is generally defined as the inability to feel pleasure in response to experiences that are usually enjoyable. Anhedonia is one of the two core symptoms of depression and is a major public health concern. Anhedonia has proven particularly difficult to counteract and predicts poor treatment response generally. It has often been hypothesized that anhedonia can be deterred by a healthy lifestyle. However, it is quite unlikely that a one-size-fits-all approach will be effective for everyone. In this study the effects of personalized lifestyle advice based on observed individual patterns of lifestyle behaviors and experienced pleasure will be examined. Further, we will explore whether a tandem skydive following the personalized lifestyle advice positively influences anhedonic young adults’ abilities to carry out the recommended lifestyle changes, and whether this ultimately improves their self-reported pleasure.

**Methods:**

Our study design is an exploratory intervention study, preceded by a cross-sectional survey as a screening instrument. For the survey, 2000 young adults (18–24 years old) will be selected from the general population. Based on survey outcomes, 72 individuals (36 males and 36 females) with persistent anhedonia (i.e., more than two months) and 60 individuals (30 males and 30 females) without anhedonia (non-anhedonic control group) will be selected for the intervention study. The non-anhedonic control group will fill out momentary assessments of pleasure and lifestyle behaviors three times a day, for one month. The anhedonic individuals will fill out momentary assessments for three consecutive months. After the first month, the anhedonic individuals will be randomly assigned to (1) no intervention, (2) lifestyle advice only, (3) lifestyle advice plus tandem skydive. The personalized lifestyle advice is based on patterns observed in the first month.

**Discussion:**

The present study is the first to examine the effects of a personalized lifestyle advice and tandem skydive on pleasure in anhedonic young adults. Results of the present study may improve treatment for anhedonia, if the interventions are found to be effective.

**Trial registration:**

Dutch Trial Register, NTR5498, registered September 22, 2015 (retrospectively registered).

**Electronic supplementary material:**

The online version of this article (doi:10.1186/s12888-016-0880-z) contains supplementary material, which is available to authorized users.

## Background

Anhedonia is generally defined as the inability to feel pleasure in response to experiences that are usually enjoyable [1]. Anhedonia is one of the two core symptoms of depression and common among young people: almost 20 % of adolescents from the general population have experienced episodes characterized by marked loss of interest in things they usually enjoy at least once in their lives [2]. Anhedonia is of major public health concern and is a dreadful experience for those who suffer from it, causing a sense of disengagement from the surrounding world and increased risk of suicide [3]. Treatment of anhedonia has received growing attention last years. It has proven particularly difficult to counteract [1] and predicts poor treatment response generally [4]. Deep brain stimulation to reward circuitry has been shown to alleviate anhedonia in treatment-resistant depressed patients [5, 6], but is too costly and invasive for large-scale application, and may lead to memory loss. Hence, we need innovative approaches to help the field forward. The challenge is to find non-invasive, non-pharmacological ways to restore a dysregulated reward system.

It is well-established that a substantial proportion of the population suffers from loss of pleasure, but less well established how exactly this loss is expressed in various aspects of life. Anhedonia is a multi-faced concept in many respects [7], and little is known about the interrelations and associated factors of these dimensions of anhedonia. To start with, individuals can experience losses in one or more different domains [8], such as food, sexual, social, and aesthetic pleasures. Heterogeneity within these domains likely arises due to the presence of different dimensions of anhedonia: consummatory versus anticipatory versus motivational anhedonia [1, 9–11]. Consummatory anhedonia concerns the pleasure experienced while experiencing a stimulus; anticipatory anhedonia involves the pleasure experienced in anticipation of a stimulus; and motivational anhedonia is about approach motivation, that is, how much effort one is willing to invest to reach a specific anticipated pleasure. So far, very little research has focused on exploring the interrelations between these different types of anhedonia.

The proposed study, which is imbedded in the project ‘*No fun no glory: a biopsychosocial investigation of how adolescents lose the ability to experience pleasure and may gain it back again’* (NWO Vici 016.001/002, granted to Prof. dr. A.J. Oldehinkel), will use state-of-the-art biopsychosocial methods to understand better what causes and sustains anhedonia, and explore the potential of a non-invasive ‘shock’ to reboot a dysregulated reward system. It has often been hypothesized that anhedonia can be deterred by a healthy lifestyle and engagement in stimulating social and physical events [12–14]. However, it is quite unlikely that a one-size-fits-all approach will be effective for everyone. As an alternative to interventions designed for typical or averaged responses, personalized medicine takes into account individual responses to potential change agents [15, 16]. Several researchers have pointed towards using ambulatory assessment as a possible tool for providing insight in daily life patterns of mood [17, 18]. Previous research in depressed patients has shown that providing feedback based on personalized patterns of positive affect is effective in decreasing depressive symptoms [19]. In our intervention study we will examine the effects of tailor-made lifestyle advice, as a non-pharmacological means to restore the pleasure of everyday activities and accomplishments, based on observed individual temporal patterns of lifestyle behaviors and experienced pleasure. Regardless of whether the advice is tailor-made or not, lifestyle changes are particularly hard to achieve or maintain for anhedonic persons, because their symptoms bereave them from the drive to pursue rewarding activities. As anhedonic persons are stuck in a spiral that is hard to break out of, mild interventions may not be effective and an intense experience may be needed to kick-start the reward system. This provocative thought will be explored in our intervention study as well, by examining whether exposure to a tandem skydive experience can help to get the reward system going again and so foster the recommended lifestyle changes that can restore the ability to experience pleasure. Thrill-experiences provide strong boosts of dopamine, and skydiving is considered an acceptable and valid human model for eliciting such responses [20, 21].

The first reason to suspect that tandem skydiving might have an effect on anhedonia is that it provokes strong emotions. Even though people rationally know that a tandem skydive is quite safe, the perception of falling down from a great height is so alarming to the brain that it elicits a fight-flight response in almost all individuals [22]. The immediate physiological response is for example reflected in increases in heart rate, blood pressure, catecholamines and cortisol levels, all of which usually return to pre-stress values not too long after the thrill experience has ended [21]. Psychologically, most people experience intense fear during the experience [22], followed by euphoria afterwards [23]. This affective contrast may be explained by Solomon’s Opponent Process Theory of Acquired Motivation, which states that humans innately contrast extreme experiences, and that removal of one emotion (in this case fear) produces the opposite response (euphoria). Another explanation for the often-reported positive feelings after a thrill experience is that conquering one’s fear boosts self-confidence and self-image [24]. These positive emotions seem an excellent start to implement life style changes that promote more persistent positive feelings.

A second reason for hypothesizing beneficial effects of tandem skydiving is that extreme experiences may shake up and reset the way how individuals respond to their environment. It is hard to predict precisely how tandem skydiving will act upon the drive to engage in pleasurable activities, because many systems are triggered in parallel. However, this is not unlike another intervention which has proven highly effective to improve mood and anhedonia in particular circumstances: electroconvulsive therapy (ECT) [25, 26]. ECT affects a wide range of neurobiological components, and numerous mechanisms have been proposed which all seem plausible [27–30]. Despite obvious differences between ECT and a tandem skydive experience, both shock experiences may set the stage for change.

In humans, not much is known about biological pathways through which experiences like tandem skydiving may induce changes in behavior and emotions. We want to address potentially relevant biological pathways in this study. By repeatedly measuring the blood markers dopamine [31, 32], serotonin [31], oxytocin [33], adrenalin, noradrenalin [34] and BDNF [27, 28, 35, 36], we will investigate specific mechanisms through which a tandem skydive accompanied by personalized lifestyle advice may help in regaining pleasure. In addition, we will assess the biomarkers BDNF and alpha-amylase around the skydive, to examine acute mechanisms of change [27, 28, 37]. Finally, previous research has shown that genes involved in the dopaminergic system, notably COMT and DAT [38, 39], as well as the BDNF gene [40] are related to reward processing, and that individuals with certain genetic variants in the DRD4 [41] and the SCL6A4 [42] gene are more susceptible for treatment. Further, animal research provided evidence that electroconvulsive therapy affects the expression of the BDNF gene, through epigenetic changes [43]. Hence, (epi)genetic factors may help explain why the intervention is or is not effective in reducing anhedonia.

In sum, the primary objectives of the current three-arm, parallel group, superiority randomized controlled trial are to examine (1) the effects of tailor-made lifestyle advices, as a non-pharmocological means to restore the pleasure in everyday activities and accomplishments and (2) whether exposure to tandem skydiving, an experience known to activate the dopamine system and to elicit strong emotions, can help to reboot the reward system and hence foster the recommended lifestyle changes. The effects of the interventions will primarily be examined with regard to experienced pleasure, but changes in blood and saliva levels of potential biomarkers are also taken into account. The secondary objectives of the current study are to (1) elucidate the various faces of pleasure loss (i.e., the different domains and dimensions) and investigate how often and in which combinations they occur among young adults, and (2) investigate relations between the various domains and dimensions of anhedonia and other factors, among which mental health problems, lifestyle behaviors, personality, self-efficacy, self-perceived social rank, and emotion recognition skills.

## Methods

### Participants

For the screening survey, 2000 young adults (18–24 years old) will be recruited from the general population. Inclusion criteria for the survey are age between 18–24 years and sufficient knowledge of the Dutch language. Inclusion criteria for the anhedonia group are (a) persistent pleasure loss and (b) willingness to perform a skydive. For the non-anhedonic control group, the inclusion criteria are (a) no loss of pleasure and (b) willingness to perform a skydive. Exclusion criteria are inability to keep an electronic diary three times a day; current professional treatment for psychiatric problems; current use of psychotropic medication; epilepsy; pregnancy; conditions that make it impossible to be attached to the tandem master (e.g., loose prostheses); height of more than 2 m; weight of more than 95 kg; inability to raise one’s legs 90° (needed for safe landing after tandem skydive); significant visual or hearing impairments; experience with skydiving, bungee jumping, base jumping, or skyjumping; cardiovascular complaints/problems. All criteria were included as questions in the screening survey.

We will use three questions from the Domains and Dimensions of Pleasure Scale ​(DDOPS; Masselink, Van Roekel, Heininga, Vrijen, Nederhof, & Oldehinkel: Domains and Dimensions of Pleasure Scale (DDOPS): a novel questionnaire to assess anhedonia, submitted) to measure persistent loss of pleasure. These questions assess the level of pleasure experienced during the past two weeks, whether this level represents a loss compared to what is considered normal to that person, and the duration of the loss. Participants who report low pleasure levels (score below the 25th percentile), a loss compared to normally experienced pleasure levels, and a minimum duration of the loss of 2 months are eligible for inclusion. We aim to complete the data collection in at least 60 anhedonic persons (20 in each condition). Because we expect some dropout during the data collection, we decided to select 24 individuals for each condition (72 in total). The non-anhedonic control group will consist of 60 individuals.

We will use stepwise inclusion for both anhedonic and non-anhedonic control participants. In the anhedonic group, we will include all participants that fulfil our criteria and are willing to participate in the study. Whenever a person is included in the anhedonic group, we will select a participant for the non-anhedonic control group, matched on sex, age, and educational level. When several control participants fulfil the criteria for matching, we will use random selection.

### Procedure

The current study will take place at the University Medical Center Groningen, the Netherlands, and uses a two-phased design. In the first phase, a survey will be administered among 2000 young adults (aged 18–24), who will be selected from secondary and higher vocational education schools in the Northern municipalities of the Netherlands, and from the University of Groningen. Participants of the survey will receive 10 Euro gift card and additionally a lottery will be held among all participants. The survey will contain, among other items, questions regarding the severity and duration of anhedonia, which will be used to select participants for the second phase of the study, i.e. the intervention part.

Individuals who are eligible for the intervention study and give permission to be contacted for further research will receive a detailed information letter and consent form by email, after which they will have one week to decide whether they want to participate. The information letter includes information about the study procedures, possible risks, insurance regulations and the possibility to stop the study at any point. Subjects who decide to participate should return a signed consent form. On the consent form, participants can indicate whether or not they give consent for storage of their body specimens for 15 years and whether or not they agree to be approached for further research in the future. Subsequently, the participants will receive an invitation for an introductory meeting during which participants’ age (checked in ID) and other exclusion criteria are discussed and checked. In addition, appointments are made and the first monthly measurements are taken.

**Fig. 1:**
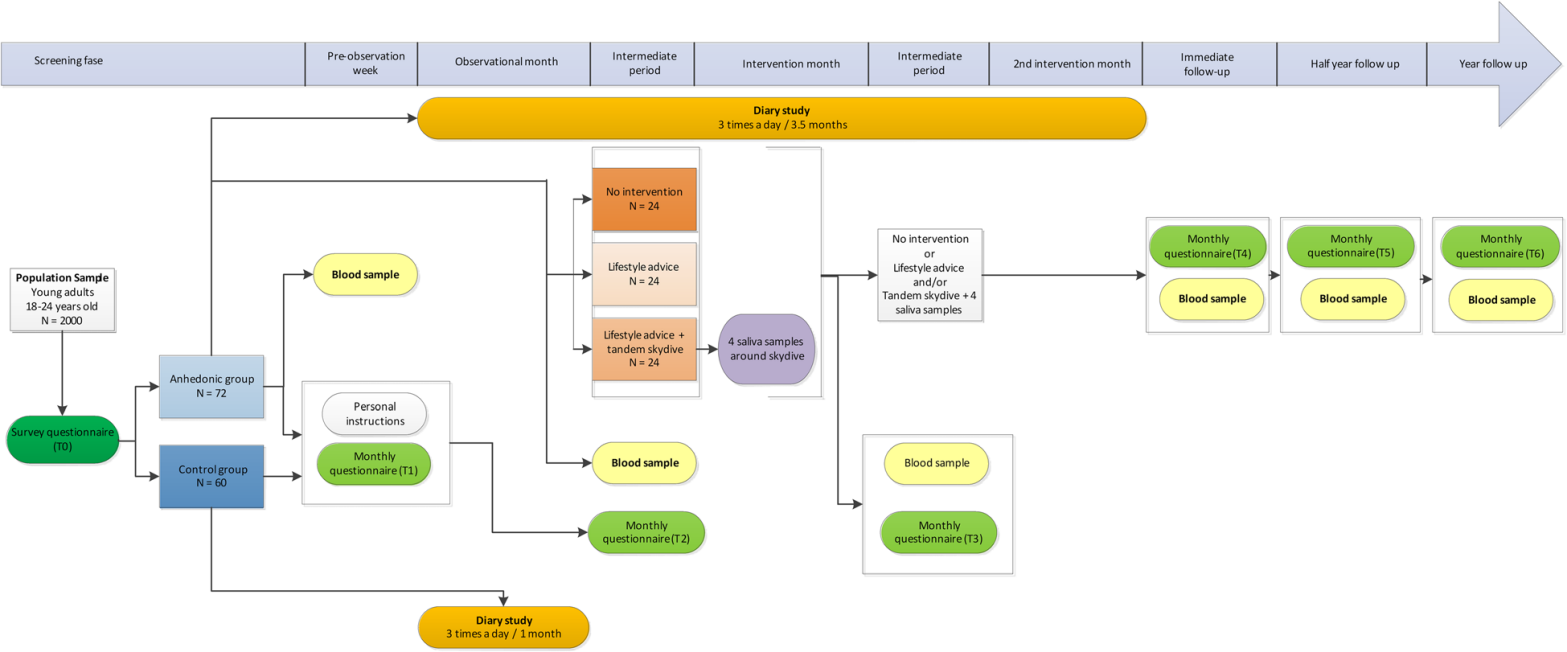
Flowchart of the study design

The intervention study consists of one pre-intervention observation month, followed by two (post-) intervention months (see Fig. 1 for a flow-chart of the intervention study). During these months, a replicated single-subject multivariate time series design will be used, in which the participants will record pleasure and possibly associated factors three times a day [44, 45], using a web-based application specifically designed for this purpose (www.roqua.nl), on their own smartphones. Time series involve a detailed collection of multiple observations within subjects [46], which allows to closely delineate processes of change over time and the temporal order of effects. The many data points and small time lags offer the opportunity to assess whether changes in one variable precede, follow, or are concurrent with changes in another, and to model potential feedback influences and cyclic patterns. As a result, inferences about the timing and direction of effects can be made more validly than in most other research designs [16, 47]. Another advantage of the small intervals between the measurements is that they reduce recall bias. Individual differences are easier to detect, as the primary analyses are done at the individual instead of the group level [16], and the design offers a powerful tool to investigate the effects of interventions [48]. Finally, the data collection takes place within participants’ natural environment, making generalization to real life more plausible [49].

After the pre-intervention observation month, the anhedonic participants will be randomly assigned (stratified on gender) to: (1) no intervention; (2) personalized lifestyle advice, based on association patterns observed in the first month; or (3) personalized lifestyle advice followed by a tandem skydive. After the second month, at the start of the third month, all participants are free to choose whatever they prefer, regardless of the intervention received after the first month: no intervention, lifestyle advice, or lifestyle advice and a tandem skydive. This last month is added to provide a more realistic impression of the feasibility and appeal of the interventions.

Before and after each observation/intervention month as well as after 6 months and one year (in total 6 times), the participants in the anhedonic groups will fill out an online questionnaire. We will collect blood samples along with the monthly and follow-up questionnaires, i.e. six times per person in total. Before and after their first skydive, participants will be asked to donate a total of four saliva samples. Saliva will be collected by means of salivettes, on which the participant has to chew. A member of the research team will provide the salivettes to the participants and provide instructions on how to use them. The salivettes will be collected by the same research member and transported to the UMCG lab.

The anhedonic intervention groups receive 75 Euro after the observation month; another 125 Euro after the first intervention month; and 200 Euro after the second intervention month. They will receive these incentives only if they reach the required compliance rate of 80% within the required time frame, if they fill out all monthly assessments and provide the required blood samples. For the two follow-up measurements they will receive 50 Euro per measurement, once assessments are filled out and blood samples are provided. This means that anhedonic participants will receive up to 500 Euro in total.

The control group of 60 non-anhedonic individuals will participate in the pre-intervention observation month only. Comparisons between this non-anhedonic control group and the intervention groups will enable us to judge whether specific daily life patterns are related to anhedonia and provide us with benchmarks with respect to the distribution and frequency of activities and associated pleasure. The non-anhedonic control group receives 75 Euro after having completed the monthly questionnaire twice and one month of momentary assessments. This fee will be conditional on the required compliance rate of 80% within the required time frame.

In order to promote retention, we will ask our participants to report any issues hampering continued participation, for example inability to fill out the diaries due to technical or practical issues (e.g., problems with internet connection, changing work schedules). We will facilitate in solutions for these issues, for example by lending mobile phones, compensating mobile internet costs and adjusting time slots for momentary assessments.

### Ethics

The present study was approved by the Medical Ethical Committee from the University Medical Center Groningen (no. 2014/508). All data received from participants will be processed in a strictly confidential manner. All participants will randomly be assigned a unique identification number, ranging from 1 to 9999. During data collection, team members will have access to the coding of the participants assigned to them in order to contact them and monitor their momentary assessments. After data collection, only the practical coordinator (TB) and the principal investigator (AO) of this study will have access to the coding of the subjects. Other investigators only have access to the coded files that do not contain information that can be traced to a specific individual. All handling of personal data will comply with the Dutch Personal Data Protection Act (Wet Bescherming Persoonsgegevens).

Although we do not expect any adverse events to occur, giving that skydiving can be considered a relatively safe sport (i.e., data from the Royal Netherlands Aeronautical Association showed no fatalities and only 0.29-0.91 minor injuries per 1000 tandem skydives in the Netherlands), we will report any serious adverse events to the medical ethical committee, according to ethical guidelines. Further, participants’ data will be monitored and if a participant’s complaints or symptoms suggest that psychiatric treatment might be indicated, we will discuss these findings with a psychiatrist in the University Center for Psychiatry, and – if the psychiatrist confirms our observation - offer that participant a consult with that psychiatrist. The study’s sponsor has an insurance to cover damage to research subjects. Further, an additional insurance covers any potential damage related to the tandem skydive.

The present study protocol was prepared in accordance with SPIRIT guidelines. The SPIRIT checklist can be found in Additional file 1.

### Randomization

Stratified by gender by means of a block randomisation procedure, participants will be randomly assigned to: (1) no intervention; (2) personalized lifestyle advice; or (3) personalized lifestyle advice followed by a tandem skydive. For males and females separately, a block size with an allocation sequence of six will be used with an equal condition distribution (i.e. each condition is present twice in a block of six). A total of 12 blocks will be created by one of the team members (MM), using an online randomisation program. The allocation will be done by other team members (i.e., ER, CV, or VH) than the one who created the allocation sequence. As it will be clear to participants which intervention they will receive, intervention allocation cannot be blinded to participants nor to the research team.

### Treatment

#### Personalized lifestyle advice

Based on diary observations during the observation month (i.e., three measures per day during 30 days) candidates will receive personalized lifestyle advice. The focus will be on lifestyle behaviors that can potentially be influenced (for exact questions, see Table 2), such as physical activities, diet, sleep, smoking and/or alcohol use, and social contacts. The advice will consist of three main parts. In the first descriptive part of the advice, general information will be presented about the participant’s average levels of lifestyle behaviors and pleasure during the observation month. In the second part, a personalized network model of the mutual relationships among momentary self-reported pleasure and lifestyle behaviors will be constructed to provide insight into temporal, potentially causal, patterns. In this part, only the lifestyle behaviors that show sufficient variability to be informative will be included. To create the personal networks, we will use Vector Autoregression (VAR) and Autoregressive Moving Average (ARMA) analyses. In the third part, we will compare participant’s average levels of lifestyle behaviors to normal population levels as observed in our own screening data as well as data from previous cohort studies in the same age group (e.g., TRAILS, [50]), in order to provide insight in plausible candidates for lifestyle changes. Based on the information presented in the previous parts, a selection will be made of the two or three lifestyle behaviors that bear the most promise for inducing pleasure, and this will be translated into a personalized lifestyle advice. Each participant will be advised to change at least two and at most three lifestyle behaviors. If possible, the advice will be based on the personalized network model described above, as this provides information about causal relationships between lifestyle behaviors and pleasure that are specific for this person. In case of too few variables in the model (i.e., too little variation in lifestyle variables) or no clear associations with pleasure, we will base the advice on the comparisons with norm levels. The advice will be discussed with the participants in person, based on a written report which participants are allowed to take home.

#### Tandem skydive

A tandem skydive is a skydive from an airplane during which the participant is securely attached to a trained tandem master. The tandem skydives will be performed from airport Hoogeveen at Skydive association Eelde-Hoogeveen. Participants do not need an extensive training; a brief instruction suffices. When they arrive at airport Hoogeveen they will first receive a brief instruction on how to keep their body in the air and how to land. Skydives will be made from an altitude of three kilometres. Landing will be in a sitting position on grass. If it turns out that participants categorically decline the tandem skydive, an alternative thrill experience will be offered. Alternatives are the ‘Skydiver’ or the ‘Goliath’, both attractions in a Dutch amusement park (i.e., Walibi Holland) that offer free fall experiences.

#### No intervention

The group receiving no intervention will continue with the momentary assessments and participate in a short meeting in which progress is checked. We chose to include an active control group that continues with momentary assessments to exclude the possibility that positive effects in the intervention groups are due to the mere registration of emotions and behaviors.

### Materials

#### Screening and monthly questionnaires

An overview of all planned monthly questionnaires can be found in Table 1. The measures in the monthly questionnaires are secondary outcome variables.

**Table 1:** Screening and Monthly Questionnaires Used in the Present Study

Concept	Questionnaire	Description	Items	Range	Authors	Assessed at:
Demographics		Questions on sex, birth date, living situation, romantic relationship, education, employment, current and lifetime psychiatric diagnosis, income	20			T0
Exclusion criteria		Questions regarding no possession of smartphone, psychiatric treatment, use of psychotropic medication, adverse conditions for skydive (e.g., epilepsy, cardiovascular problems, weight, height, pregnancy), experience with skydive	16			T0-T6
Reward responsiveness	RR	Questionnaire assessing reward responsiveness	8	1–4	Van den Berg, Franken, & Muris, 2010 [56]	T0-T6
Pleasure and pleasure loss	DDOPS	Questionnaire assessing pleasure in different domains (i.e., perceptual, social, sexual and personal achievement) and different dimensions (i.e., consummatory, anticipatory, motivational)	31 pleasure level items, 31 pleasure change items	1–100 for level of pleasure, 1–5 for change in pleasure	Masselink et al., submitted	T0-T6
Lifestyle factors	TRAILS lifestyle	Questionnaire assessing the frequency of and amount of time spent on various lifestyle-related activities in a typical week (e.g., traveling to work, hobbies, leisure activities, exercise).	27 + 3–4 extra items per endorsed activity	Yes/no + frequency + time	Ormel et al., 2012 [57]	T0-T6
Substance use	TRAILS substance use	Questionnaire assessing the frequency and amount of smoking, alcohol, soft drugs and hard drugs.	4–12	Differs per question		T0-T6
Life events and difficulties	TRAILS + own items	Questionnaire assessing chronic difficulties (e.g., long lasting conflict, chronic disease or handicap, unemployment) and recent life events (i.e., relationship break-up, failing to reach important goal, death of loved one).	13	Yes/no	Ormel et al., 2012 [57]	T0-T6
Depressive symptoms	PHQ-9	Questionnaire assessing depressive symptoms in the past two weeks, based on the DSM-IV criteria for depression.	9	0-3	Kroenke, Spitzer, & Williams, 2001 [58]	T0-T6
Psychopathology	ASR	Questionnaire assessing various mental health problems in the past six months.	123	0–2	Achenbach & Rescorla, 2001 [59]	T0, T5, T6
Positive affect	PA	Questionnaire assessing general positive affect, which was partly based on previous research and partly newly constructed.	11		Wichers et al., 2007; Wichers, Lothmann, Simons, Nicolson, & Peeters, 2012 [60, 61]	T0-T6
Effortful control	ATQ-EC	Questionnaire assessing activation control, attentional control and inhibitory control.	19	1–7	Evans & Rothbart, 2007 [62]	T0, T4
Extraversion	BFI-E	The extraversion subscale from the Big Five Inventory.	8	1–5	Denissen, Geenen, van Aken, Gosling, & Potter, 2008 [63]	T0
Self-efficacy	GSS	Questionnaire assessing general self-efficacy.	10	1–4	Teeuw, Schwarzer, & Jerusalem, 1994 [64]	T0-T6
Social comparison	SCS	Questionnaire assessing different aspects of social comparison: judgments with regard to social rank, social attractiveness, and social group fit.	10	1–10	Allan & Gilbert, 1995 [65]	T0-T6
Consummatory and anticipatory pleasure	TEPS	Questionnaire assessing the general level of consummatory (9 items) and anticipatory pleasure (9 items).	18	1–6	Gard, Gard, Kring, & John, 2006 [66]	T0
Facial emotion recognition	FER morphing task	Task consisting of 24 video clips in which a neutral face morphs into a face with an emotional expression of anger, fear, sadness or happiness. Assesses average reaction time (i.e., speed) and number of correctly identified emotions (i.e., accuracy).	24	n/a	Heuer, Lange, Isaac, Rinck, & Becker, 2010 [67]	T0, T2, T3, T4

#### Momentary questionnaires

The items for the momentary questionnaires are presented in Table 2. Pleasure is the primary outcome measure. The other momentary items relate to secondary outcome variables.

**Table 2:** Description of Momentary Questionnaire

Question	Dutch	Translation	Response range	Range	Description
1.	Ik probeerde in slaap te vallen om…	I tried to fall asleep at…	Hours - minutes	n/a	Time of trying to sleep
2.	Aantal minuten totdat ik in slaap viel	Number of minutes until falling asleep	Number of minutes	n/a	Time until falling asleep
3.	Ik werd wakker om…	I woke up at…	Hours - minutes	n/a	Time of waking up
4.	Hoeveel keer werd ik wakker gedurende de nacht?	How many times did I wake up during the night?	0, 1, 2, 3, 4, 5 or more	0–5	Frequency of awakenings
5.	Totaal aantal minuten wakker geweest	Total number of minutes awake	Number of minutes		Time of awakenings
6.	Mijn slaapkwaliteit was	My sleep quality was	“Very bad” to “Very good”	0–100	Sleep quality
7.	Ik heb vervelend gedroomd	I had bad dreams	“Not at all” to “Very much”	0–100	Nightmares
8.	Ik voelde me uitgerust bij het wakker worden	I felt rested when I woke up	“Not at all” to “Very much”	0–100	Rest
9.	Ik ben nu…	I am now…	(1) Home (2) Work/school (3) On the way (4) Restaurant/café (5) In nature (6) Vacation home/hotel/camping (7) Hospital/care facility (8) Shop (9) Gym/sports club (10) Somewhere else	1–10	Current location (only one box could be checked)
10.	Ik ben nu…	I am now…	(1) Alone (2) With partner (3) With family (4) With friends (5) With fellow students (6) With colleagues (7) With acquaintances (8) With strangers	1–8	Current social context (multiple answers possible)
11.	Ik voel me nu plezierig	I feel pleasurable	“Not at all” to “Very much”	0–100	Current pleasure
12.	Ik voelde me geïnteresseerd in de dingen om me heen	I felt interested in the things around me	“Not at all” to “Very much”	0–100	Positive affect, low arousal
13.	Ik voelde me overstuur	I felt upset	“Not at all” to “Very much”	0–100	Negative affect, high arousal
14.	Ik voelde me opgewekt	I felt joyful	“Not at all” to “Very much”	0–100	Positive affect, high arousal
15.	Ik voelde me somber	I felt gloomy	“Not at all” to “Very much”	0–100	Negative affect, low arousal
16.	Ik voelde me daadkrachtig	I felt determined	“Not at all” to “Very much”	0–100	Positive affect, high arousal
17.	Ik was niet vooruit te branden	I felt sluggish	“Not at all” to “Very much”	0–100	Negative affect, low arousal
18.	Ik voelde me kalm	I felt calm	“Not at all” to “Very much”	0–100	Positive affect, low arousal
19.	Ik voelde me angstig	I felt anxious	“Not at all” to “Very much”	0–100	Negative affect, high arousal
20.	Ik voelde me levenslustig	I felt lively	“Not at all” to “Very much”	0–100	Positive affect, high arousal
21.	Ik voelde me verveeld	I felt bored	“Not at all” to “Very much”	0–100	Negative affect, low arousal
22.	Ik voelde me enthousiast	I felt enthusiastic	“Not at all” to “Very much”	0–100	Positive affect, high arousal
23.	Ik voelde me geirriteerd	I felt irritated	“Not at all” to “Very much”	0–100	Negative affect, high arousal
24.	Ik voelde me ontspannen	I felt relaxed	“Not at all” to “Very much”	0–100	Positive affect, low arousal
25.	Ik voelde me vrolijk	I felt cheerful	“Not at all” to “Very much”	0–100	Positive affect, high arousal
26.	Ik voelde me nerveus	I felt nervous	“Not at all” to “Very much”	0–100	Negative affect, high arousal
27.	Ik voelde me tevreden	I felt content	“Not at all” to “Very much”	0–100	Positive affect, low arousal
28.	Ik voelde me lusteloos	I felt listless	“Not at all” to “Very much”	0–100	Negative affect, low arousal
29.	Ik voelde me energiek	I felt energetic	“Not at all” to “Very much”	0–100	Positive affect, high arousal
30.	Ik heb sinds de laatste meting plezier gehad	I experienced pleasure since the last assessment	“Not at all” to “Very much”	0–100	Pleasure since the last assessment
31.	Ik heb gepiekerd	I have been worrying	“Not at all” to “Very much”	0–100	Worrying
32.	Ik was tevreden over mezelf	I was pleased with myself	“Not at all” to “Very much”	0–100	Self-esteem
33.	Waar was ik sinds de vorige meting?	Where was I since the last assessment?	(1) Home (2) Work/school (3) On the way (4) Restaurant/café (5) In nature (6) Vacation home/hotel/camping (7) Hospital/care facility (8) Shop (9) Gym/sports club (10) Somewhere else	1–10	Location since last assessment (multiple answers possible). In case of multiple locations, go to 34.
34.	Waar heb ik de meeste tijd doorgebracht?	Where have I spent most time?	Only locations checked in previous question		Main location (only one answer possible).
35.	Ik ben sinds de laatste meting buiten geweest	I have been outside since the last assessment	“Not at all” to “Very much”	0–100	Being outside
36.	Ik was sinds de laatste meting	Since the last assessment, I was	(1) Alone (2) With partner (3) With family (4) With friends (5) With fellow students (6) With colleagues (7) With acquaintances (8) With strangers	1–8	Social context since last assessment (multiple answers possible). All checked answers were included in next question
36. a-x	Tijd die ik met [gezelschap uit vraag 36] heb doorgebracht	Time spent with [company from question 36]	“Very little” to “Very much”	0–100	Time spent in social context
37.	Hoeveel heb ik gepraat met andere mensen?	How much have I been talking to other people?	“Not at all” to “Very much”	0–100	Social interaction
38.	Ik heb sinds de laatste meting de volgende activiteiten gedaan:	I participated in the following activities since the last assessment:	(1) Watching TV (2) Listening to music (3) Studying (4) Reading a book or magazine (5) Shopping (6) Using the internet (7) Gaming (8) Working (9) Having sex (alone or with someone else) (10) Household chores (11) Sports (12) Going to a bar or club (13) Going out for dinner (14) Going to the movies, theatre, museum or concert (15) Sleeping (16) Using social media (17) Hobbies (18) Other, namely….	1–18	Activities (multiple answers possible)
39.	Ik ben sinds de laatste meting lichamelijk actief geweest	I have been physically active since the last assessment	“Not at all” to “Very much”	0–100	
40.	Ik heb sinds de laatste meting de volgende middelen gebruikt:	I used the following substances since the last assessment:	(1) Nothing (2) Caffeine (3) Nicotine (4) Medication (5) Alcohol (6) Cannabis (7) Stimulants (8) Sedatives (9) Other drugs	1–9	Substance use (if caffeine, nicotine, or alcohol was checked, go to question 41)
41.	Aantal [cafeine-houdende drankjes/sigaretten/glazen alcohol] dat ik sinds de laatste meting heb [gedronken/gerookt/gedronken]	Number of [caffeine-containing drinks/cigarettes/glasses of alcohol] consumed since the last assessment	Number	n/a	Amount of substance use
42.	Ik heb sinds de laatste meting het volgende gegeten:	I have eaten the following since the last assessment:	(1) Nothing (2) Dairy products (3) Vegetables/fruit (4) Bread/pasta/rice/potatoes (5) Meat (6) Fish (7) Eggs (8) Nuts (9) Meat or dairy substitutes	1–9	Food intake
43.	Hoeveel zoete snacks (bijv. koek, snoep) heb ik gegeten?	How many sweet snacks (e.g., cookies, sweets) did I eat?	“Nothing” to “A lot”	0–100	Sweet snacks
44.	Hoeveel hartige snacks (bijv. patat, chips) heb ik gegeten?	How many savory snacks (e.g., French fries, potato chips) did I eat?	“Nothing” to “A lot”	0–100	Savory snacks
45.	Denk aan de leukste gebeurtenis die je hebt meegemaakt sinds de laatste meting: hoe plezierig was deze gebeurtenis voor jou?	Think about the most pleasant event you experienced since the last assessment: how enjoyable was this experience?	“Not at all” to “Very much”	0–100	Pleasantness most positive event
46.	Denk aan de leukste gebeurtenis die je hebt meegemaakt sinds de laatste meting: hoe belangrijk was deze gebeurtenis voor jou?	Think about the most pleasant event you experienced since the last assessment: how important was this experience?	“Not at all” to “Very much”	0–100	Importance most positive event
47.	Denk aan de vervelendste gebeurtenis die je hebt meegemaakt sinds de laatste meting: hoe onplezierig was deze gebeurtenis voor jou?	Think about the most unpleasant event you experienced since the last assessment: how unpleasant was this event?	“Not at all” to “Very much”	0–100	Unpleasantness most negative event
48.	Denk aan de vervelendste gebeurtenis die je hebt meegemaakt sinds de laatste meting: hoe belangrijk was deze gebeurtenis voor jou?	Think about the most unpleasant event you experienced since the last assessment: how important was this event?	“Not at all” to “Very much”	0–100	Importance most negative event
49.	Ik heb sinds de laatste meting lichamelijk ongemak ervaren (hoofdpijn, diarree, zware benen, etc.)	I have experienced physical discomfort since the last assessments (head ache, diarrhea, heavy legs, etc.)	“Not at all” to “Very much”	0–100	Physical discomfort
50.	Sinds de laatste meting had ik het:	How busy was I since the last assessment:	(1) Much too busy (2) Too busy (3) Pleasantly busy (4) Neutral (5) Pleasantly quiet (6) Too quiet (7) Much too quiet	1–7	Time pressure/stress

*Note.* The affect items (i.e., item 12–29) were assessed about the current moment in the morning assessment, and about the time since the last assessment in the afternoon and evening assessments. These items are based on Russell’s circumplex model of affect [68], including items for high and low arousal PA and NA, and we created some additional items to measure pleasure (items 11, 30), interest (item 12), and motivation (items 16, 17, 28). The sleep items (items 1–8) were based on the Pittsburgh sleep diary [69]. Items for location (items 9, 33), social context (items 10, 36, 36a-x), worrying (item 31), self-esteem (item 32), being outside (item 35), social interaction (item 37), activities (item 38), physical activity (item 39), positive and negative events (items 45–48), physical discomfort (item 49), and time pressure (item 50) were adapted from previous ESM studies [55, 70–73]. We created new items to assess substance use (items 40–41), food intake (item 42), and sweet and savory snacks (items 43, 44)

#### Blood samples

The collected blood samples will be used to measure levels of dopamine, serotonin, oxytocin, adrenalin, noradrenalin, and brain-derived neurotrophic factor (BDNF). Part of the first blood sample will be stored to allow later assessment of polymorphisms in relevant candidate genes; and part of the first, third, fifth and sixth blood sample will be stored to allow later epigenetic analyses. As mentioned in the introduction, (epi-) genetic factors are assumed to play an essential role in mechanisms explaining why interventions are or are not effective in reducing symptoms. We will examine genetic polymorphisms in several genes (i.e., DRD2, DRD4, DAT, COMT, 5-HTTLPR, BDNF). Further, we will examine epigenetic modifications in genes in the dopaminergic system and in the BDNF gene. Given the relatively small sample size, power to detect (epi-) genetic effects is probably insufficient with the current (epi-) genetic analyses techniques. However, analytic techniques improve rapidly, allowing smaller sample sizes. We will only conduct these (epi-) genetic analyses when the appropriate techniques and analyses are available, and the statistical power is sufficient to allow meaningful conclusions. At each of the time points, the blood samples will consist of one 10 ml EDTA K2E tube and, dependent on time point, one or two 4 ml EDTA K2E tube(s).

#### Saliva samples

We will assess the biomarker alpha-amylase in saliva around the skydive, to examine acute mechanisms of change [20, 37].

### Sample size calculation

The sample size of the survey will be at least *N* = 2000. Estimates from a pilot study conducted in 277 young adults, using the same criteria as will be used in the present study, showed that around 5 % of the sample experienced persistent anhedonia. Hence, a sample size of 2000 will yield *N* = 100 individuals with persistent anhedonia, which will warrant sufficient power to investigate relationships among different dimensions and domains of anhedonia and between these dimensions and domains and other relevant factors.

In the intervention study, we aim to collect time series data in at least 60 (72 including oversampling) anhedonic persons. With regard to our main research question, that is, whether the different interventions lead to improvements in pleasure scores, we will follow two approaches. First, following from the exploratory nature of the intervention study, we will examine each individual series of observations to assess individual response patterns. This will be done by means of (interrupted) times series analysis (ITS) to detect time trends in the amount of pleasure experienced during the study, and differences in the level and slope between pre- and post- intervention pleasure levels. It is not well possible to calculate the power of time series analyses because that depends on a wide range of factors, but a general recommendation is that there should be at least 12 observations before and 12 after the intervention [51]. Considering that we have at least 90 observations before and after the interventions, we do not foresee any power problems.

To explore patterns of change at a group level, we will conduct multilevel analyses with pleasure level after the intervention as outcome variable. In these analyses, Level 1 represents the assessment level (i.e., momentary assessments within individuals) and Level 2 the individual level. In order to be able to examine cross-level interactions (i.e., testing whether Level 1 relations differ between individuals), a minimum number of 50 Level 2 cases and 20 Level 1 cases should be sufficient [52]. As we include at least 60 individuals (Level 2) and 90 momentary assessments (Level 1), we should have sufficient power to answer our main research question.

Finally, to explore patterns of change in monthly assessed questionnaires, we will use repeated measures ANOVAs. A power analyses in Stata showed that we need 21 individuals per group to detect a large difference between groups (Cohen’s d = 0.8) with a correlation of 0.6 between the repeated assessments (power = 0.8, alpha = 0.05). As we include 24 participants per group (oversampled for drop-out), we should have enough power to detect large differences.

### Statistical analyses

#### Primary outcome variables

First, we will examine individual response patterns by means of (interrupted) time series analysis (ITS) to detect time trends in the amount of pleasure experienced during the study, and differences in the level and slope between pre- and post-intervention pleasure levels. Interrupted time series analysis is particularly useful to test changes in outcome rates in time periods before and after implementation of an intervention designed to change the outcome (see, for instance, [53]). The number of individuals who show a significant improvement in pleasure in the second month of the study period as compared to the first one will be compared across the three intervention groups to assess to what extent this improvement, if any, is likely to be due to spontaneous recovery or the mere daily registration of pleasure and activities, to the personalized lifestyle advice, or to the tandem skydive experience. In addition, we will explore whether changes in pleasure scores from pre- to post-intervention differ between the three intervention groups in a multilevel model. We will compare both (a) individuals who did and did not receive lifestyle advice; and, within the group who received lifestyle advice, (b) individuals who did and did not perform a skydive. We will use a multilevel model with two levels, in which Level 1 represents the momentary assessments, and Level 2 the individual level (e.g., intervention group). The main advantage of multilevel analyses is that it takes the nested structure of the data into account. By conducting multilevel analyses, we will be able to compare post-intervention mean levels of momentary pleasure between the three intervention groups, while controlling for the level of pleasure in the pre-intervention period (i.e., the observation month).

### Additional analyses

Data collected in the present study will provide ample opportunities to answer many different research questions. It is not possible to describe all potential research questions in detail, but we will highlight some of them here. First, the above-described procedure relates to pleasure scores but a similar approach can be followed to investigate changes in specific lifestyle behaviors, to answer the question to what extent each of the interventions induced health-related behavioral change.

Second, another goal with regard to the secondary study parameters is to examine whether the levels of specific biomarkers (e.g., dopamine, BDNF) are associated with the momentary pleasure levels. This will be investigated by conducting multilevel analyses with three levels: the assessment level (Level 1), the month level (Level 2), and the individual level (Level 3). In this way, we can explore how levels of these specific biomarkers are cross-sectionally and prospectively associated with momentary pleasure levels and changes therein. This design will allow to examine whether and how the level of biomarkers measured at the beginning of a month are predictive for the momentary pleasure levels experienced during that month.

Finally, the survey data (*N* = 2000) can be used in order to elucidate the various faces of pleasure loss (i.e., in different domains and dimensions), and how often and in which combinations they occur among young adults. This will be done by conducting latent class analysis. In latent class analysis, it is possible to examine whether individuals can be grouped according to their pattern of pleasure loss. Further, when different groups of individuals are found, we will be able to predict group membership, by examining whether different variables increase the odds of being in a particular anhedonia group.

### Missing data

Participants are required to complete at least 80 % of the momentary assessments each month to be included in the analyses. Participants who complete less than 80 % in the first month, will be excluded from the study. Attrition analyses will be conducted to compare these drop-outs with the participants on demographic characteristics and levels of pleasure. Missing data will be imputed by means of Expectation-Maximization imputation.

## Discussion

This study protocol describes a two-phase exploratory intervention study, in which the effects of personalized lifestyle advices and tandem skydives are examined. We hypothesize that providing anhedonic young adults with a personalized lifestyle advice will increase their pleasure levels. Further, we hypothesize that a tandem skydive can reboot the reward system, which would lead to further increases in pleasure, above the effects of the lifestyle advice.

### Strengths and limitations

The study has several strengths. First, it is the first study to explore the effects of a personalized lifestyle advice and a tandem skydive, and hence can provide new insights in effective treatments for anhedonia. Second, as we assess pleasure and lifestyle behaviors in real life and the advices are tailor-made, we ensure that the advices are relevant to each individual. Third, the long period of momentary assessments (i.e., three months) will enable us to explore individual differences in the effects of the interventions and to examine whether differences between groups are due to non-specific intervention effects (e.g., an upward trend that was already present in the observation period). Fourth, by using a randomized design, we will be able to compare whether the lifestyle advice and tandem skydive are more effective in increasing pleasure than only registering one’s emotions and daily life activities.

Despite these strengths, there are also limitations. First, although we follow general recommendations with regard to sample size, we may not be able to detect subtle differences between groups. However, because of the large number of momentary assessments we will be able to detect differences between groups on momentarily assessed outcome variables, and our individual analyses will enable us to determine whether further examination of these interventions in larger samples is likely to be fruitful. Second, as we need to include participants who may be willing to perform a tandem skydive, the generalization of our findings may be limited. We will check in the analyses whether this is indeed the case, by comparing the group of anhedonic individuals that is willing to perform a skydive with the group that is not willing to perform a skydive on demographic characteristics and main study variables. Third, although including more than three measures per day is not feasible due to the long duration of the study (i.e., three months), we might miss some information about lifestyle behaviors and associated pleasure due to the 6 h intervals between assessments. If the intervention proves to be effective, further research examining the effects of lifestyle advices could include more assessments per day, over a short time span (e.g., one month).

### Clinical implications

Findings from the present study could lead to improved treatment for anhedonia. As recent research indicates that it is possible to automate personalized feedback [54, 55], the proposed intervention could be easily implemented in clinical settings, if proven effective. The results from the individual models could lead to additional insights in what works for whom, which could provide starting points for personalizing treatment. Further, if the tandem skydive is found to be effective and pending replication of these potential positive effects, a tandem skydive could be considered as an addition to treatment-as-usual.

## Additional file

Additional file 1: SPIRIT checklist. (DOC 120 kb)

## References

[1] Treadway MT, Zald DH (2011). Reconsidering anhedonia in depression: lessons from translational neuroscience. Neurosci Biobehav Rev.

[2] Bennik EC, Nederhof E, Ormel J, Oldehinkel AJ (2014). Anhedonia and depressed mood in adolescence: course, stability, and reciprocal relation in the TRAILS study. Eur Child Adolesc Psychiatry.

[3] Bolton JM, Pagura J, Enns MW, Grant B, Sareen J (2010). A population-based longitudinal study of risk factors for suicide attempts in major depressive disorder. J Psychiatr Res.

[4] Spijker J, Bijl RV, De Graaf R, Nolen WA (2001). Determinants of poor 1-year outcome of DSM-III-R major depression in the general population: results of the Netherlands Mental Health Survey and Incidence Study (NEMESIS). Acta Psychiatr Scand.

[5] Giacobbe P, Kennedy SH (2006). Deep brain stimulation for treatment-resistant depression: a psychiatric perspective. Curr Psychiatry Rep.

[6] Schlaepfer TE, Cohen MX, Frick C, Kosel M, Brodesser D, Axmacher N (2007). Deep brain stimulation to reward circuitry alleviates anhedonia in refractory major depression. Neuropsychopharmacology.

[7] Gruzelier J, Davis S (1995). Social and physical anhedonia in relation to cerebral laterality and electrodermal habituation in unmedicated psychotic patients. Psychiatry Res.

[8] Chapman LJ, Chapman JP, Raulin ML (1976). Scales for physical and social anhedonia. J Abnorm Psychol.

[9] Germans MK, Kring AM (2000). Hedonic deficit in anhedonia: support for the role of approach motivation. Personal Individ Differ.

[10] Shankman SA, Klein DN, Tenke CE, Bruder GE (2007). Reward sensitivity in depression: a biobehavioral study. J Abnorm Psychol.

[11] Sherdell L, Waugh CE, Gotlib IH (2012). Anticipatory pleasure predicts motivation for reward in major depression. J Abnorm Psychol.

[12] Rasmussen M, Laumann K (2014). The role of exercise during adolescence on adult happiness and mood. Leis Stud.

[13] Schuster TL, Kessler RC, Aseltine RH (1990). Supportive interactions, negative interactions, and depressed mood. Am J Community Psychol.

[14] Stubbe JH, de Moor MHM, Boomsma DI, de Geus EJC (2007). The association between exercise participation and well-being: a co-twin study. Prev Med.

[15] Ahn AC, Tewari M, Poon C-S, Phillips RS (2006). The limits of reductionism in medicine: could systems biology offer an alternative?. PLoS Med.

[16] Molenaar PCM, Campbell CG (2009). The new person-specific paradigm in psychology. Curr Dir Psychol Sci.

[17] Ebner-Priemer UW, Trull TJ (2009). Ambulatory assessment: an innovative and promising approach for clinical psychology. Eur Psychol.

[18] Wichers M, Simons CJP, Kramer IMA, Hartmann JA, Lothmann C, Myin-Germeys I (2011). Momentary assessment technology as a tool to help patients with depression help themselves. Acta Psychiatr Scand.

[19] Kramer I, Simons CJP, Hartmann JA, Menne-Lothmann C, Viechtbauer W, Peeters F (2014). A therapeutic application of the experience sampling method in the treatment of depression: a randomized controlled trial. World Psychiatry.

[20] Chatterton RT, Vogelsong KM, Lu Y, Hudgens GA (1997). Hormonal responses to psychological stress in men preparing for skydiving ^1^. J Clin Endocrinol Metab.

[21] van Westerloo DJ, Choi G, Lowenberg EC, Truijen J, de Vos AF, Endert E (2011). Acute stress elicited by bungee jumping suppresses human innate immunity. Mol Med.

[22] Alpers GW, Adolph D (2008). Exposure to heights in a theme park: fear, dizziness, and body sway. J Anxiety Disord.

[23] Celsi RL, Rose RL, Leigh TW (1993). An exploration of high-risk leisure consumption through skydiving. J Consum Res.

[24] Brymer E, Schweitzer R (2013). Extreme sports are good for your health: a phenomenological understanding of fear and anxiety in extreme sport. J Health Psychol.

[25] American Psychiatric Association. The Practice of Electroconvulsive Therapy: Recommendations for Treatment, Training, and Privileging (A Task Force Report of the American Psychiatric Association). Washington, DC: American Psychiatric Association; 2008.

[26] Nutt DJ, Baldwin DS, Clayton AH. The role of dopamine and norepinephrine in depression and antidepressant treatment. J. Clin. Psychiatry. 2006;67:3-8. 16848670

[27] Bocchio-Chiavetto L, Zanardini R, Bortolomasi M, Abate M, Segala M, Giacopuzzi M (2006). Electroconvulsive Therapy (ECT) increases serum Brain Derived Neurotrophic Factor (BDNF) in drug resistant depressed patients. Eur Neuropsychopharmacol.

[28] Marano CM, Phatak P, Vemulapalli UR, Sasan A, Nalbandyan MR, Ramanujam S (2007). Increased plasma concentration of brain-derived neurotrophic factor with electroconvulsive therapy: a pilot study in patients with major depression. J Clin Psychiatry.

[29] Palmio J, Huuhka M, Saransaari P, Oja SS, Peltola J, Leinonen E (2005). Changes in plasma amino acids after electroconvulsive therapy of depressed patients. Psychiatry Res.

[30] Schmidt EZ, Reininghaus B, Enzinger C, Ebner C, Hofmann P, Kapfhammer HP (2008). Changes in brain metabolism after ECT–Positron emission tomography in the assessment of changes in glucose metabolism subsequent to electroconvulsive therapy — Lessons, limitations and future applications. J Affect Disord.

[31] Der-Avakian A, Markou A (2012). The neurobiology of anhedonia and other reward-related deficits. Trends Neurosci.

[32] Wise RA (2004). Dopamine, learning and motivation. Nat Rev Neurosci.

[33] Bell CJ, Nicholson H, Mulder RT, Luty SE, Joyce PR (2006). Plasma oxytocin levels in depression and their correlation with the temperament dimension of reward dependence. J Psychopharmacol (Oxf).

[34] Kuffel A, Eikelmann S, Terfehr K, Mau G, Kuehl LK, Otte C (2014). Noradrenergic blockade and memory in patients with major depression and healthy participants. Psychoneuroendocrinology.

[35] Castrén E, Võikar V, Rantamäki T (2007). Role of neurotrophic factors in depression. Curr Opin Pharmacol.

[36] Duman RS, Monteggia LM (2006). A neurotrophic model for stress-related mood disorders. Biol Psychiatry.

[37] Tanaka Y, Ishitobi Y, Maruyama Y, Kawano A, Ando T, Okamoto S (2012). Salivary alpha-amylase and cortisol responsiveness following electrical stimulation stress in major depressive disorder patients. Prog Neuropsychopharmacol Biol Psychiatry.

[38] Dreher J-C, Kohn P, Kolachana B, Weinberger DR, Berman KF (2009). Variation in dopamine genes influences responsivity of the human reward system. Proc Natl Acad Sci.

[39] Hahn T, Heinzel S, Dresler T, Plichta MM, Renner TJ, Markulin F (2011). Association between reward-related activation in the ventral striatum and trait reward sensitivity is moderated by dopamine transporter genotype. Hum Brain Mapp.

[40] Peciña M, Martínez-Jauand M, Love T, Heffernan J, Montoya P, Hodgkinson C (2014). Valence-specific effects of BDNF Val66Met polymorphism on dopaminergic stress and reward processing in humans. J Neurosci.

[41] Bakermans-Kranenburg MJ, Van IMH, Pijlman FT, Mesman J, Juffer F (2008). Experimental evidence for differential susceptibility: dopamine D4 receptor polymorphism (DRD4 VNTR) moderates intervention effects on toddlers’ externalizing behavior in a randomized controlled trial. Dev Psychol.

[42] Serretti A, Kato M, De Ronchi D, Kinoshita T (2006). Meta-analysis of serotonin transporter gene promoter polymorphism (5-HTTLPR) association with selective serotonin reuptake inhibitor efficacy in depressed patients. Mol Psychiatry.

[43] Tsankova NM, Kumar A, Nestler EJ (2004). Histone modifications at gene promoter regions in rat hippocampus after acute and chronic electroconvulsive seizures. J Neurosci.

[44] Jacobs N, van Os J, Derom C, Thiery E, Delespaul P, Wichers M (2011). Neuroticism explained? From a non-informative vulnerability marker to informative person–context interactions in the realm of daily life. Br J Clin Psychol.

[45] Wichers M, Aguilera M, Kenis G, Krabbendam L, Myin-Germeys I, Jacobs N (2007). The catechol-O-methyl transferase Val158Met polymorphism and experience of reward in the flow of daily life. Neuropsychopharmacology.

[46] Chatfield C. The Analysis of Time Series: An Introduction, Sixth Edition. London: Chapman & Hall/CRC; 2004.

[47] Brandt PT, Williams JT. Multiple Time Series Models. Thousand Oaks, CA: SAGE; 2007.

[48] Kratochwill TR, Levin JR (1992). Single-case research design and analysis: new directions for psychology and education.

[49] Hilliard RB (1993). Single-case methodology in psychotherapy process and outcome research. J Consult Clin Psychol.

[50] Huisman M, Oldehinkel AJ, De Winter AF, Minderaa RB, de Bildt A, Huizink AC, et al. Cohort profile: The Dutch “TRacking Adolescents” Individual Lives’ Survey’’; TRAILS. Int J Epidemiol. 2008;37:1227–35. 10.1093/ije/dym273 18263649

[51] Wagner AK, Soumerai SB, Zhang F, Ross-Degnan D (2002). Segmented regression analysis of interrupted time series studies in medication use research. J Clin Pharm Ther.

[52] Hox JJ. Multilevel Analysis: Techniques and Applications. Mahwah, NJ: Lawrence Erlbaum Associates Inc.; 2002.

[53] Penfold RB, Zhang F (2013). Use of interrupted time series analysis in evaluating health care quality improvements. Acad Pediatr.

[54] van der Krieke L, Jeronimus BF, Blaauw FJ, Wanders RBK, Emerencia AC, Schenk HM, et al. HowNutsAreTheDutch (HoeGekIsNL): A crowdsourcing study of mental symptoms and strengths. Int. J Methods Psychiatr Res. 2015;1–22. doi:10.1002/mpr.1495. 10.1002/mpr.1495 PMC6877205 26395198

[55] Emerencia A, van der Krieke L, Bos E, de Jonge P, Petkov N, Aiello M (2015). Automating vector autoregression on electronic patient diary data. IEEE J Biomed Health Inform.

[56] Van den Berg I, Franken IHA, Muris P. A new scale for measuring reward responsiveness. Front Psychol. 2010;1:1-7. 10.3389/fpsyg.2010.00239 PMC3153843 21922010

[57] Ormel J, Oldehinkel AJ, Sijtsema J, van Oort F, Raven D, Veenstra R (2012). The TRacking Adolescents’ Individual Lives Survey (TRAILS): design, current status, and selected findings. J Am Acad Child Adolesc Psychiatry.

[58] Kroenke K, Spitzer RL, Williams BW (2001). The PHQ-9. J Gen Intern Med.

[59] Achenbach TM, Rescorla LA (2001). Manual for the ASEBA school-age forms and profiles: an integrated system of multi-informant assessment.

[60] Wichers M, Aguilera M, Kenis G, Krabbendam L, Myin-Germeys I, Jacobs N, et al. The Catechol-O-Methyl transferase Val158Met polymorphism and experience of reward in the flow of daily life. Neuropsychopharmacology. 2007;1–7. 10.1038/sj.npp.1301520 17687265

[61] Wichers M, Lothmann C, Simons CJP, Nicolson NA, Peeters F (2012). The dynamic interplay between negative and positive emotions in daily life predicts response to treatment in depression: a momentary assessment study. Br J Clin Psychol.

[62] Evans DE, Rothbart MK (2007). Developing a model for adult temperament. J Res Personal.

[63] Denissen JJA, Geenen R, van Aken MAG, Gosling SD, Potter J (2008). Development and validation of a Dutch translation of the Big Five Inventory (BFI). J Pers Assess.

[64] Teeuw B, Schwarzer R, Jerusalem M. Dutch adaptation of the general self-efficacy scale. Berlin; 1994. http://userpage.fu-berlin.de/~health/dutch.htm.

[65] Allan S, Gilbert P (1995). A social comparison scale: psychometric properties and relationship to psychopathology. Personal Individ Differ.

[66] Gard DE, Gard MG, Kring AM, John OP (2006). Anticipatory and consummatory components of the experience of pleasure: a scale development study. J Res Personal.

[67] Heuer K, Lange W-G, Isaac L, Rinck M, Becker ES (2010). Morphed emotional faces: emotion detection and misinterpretation in social anxiety. J Behav Ther Exp Psychiatry.

[68] Russell JA (1980). A circumplex model of affect. J Pers Soc Psychol.

[69] Monk TH, Reynolds CF, Kupfer DJ, Buysse DJ, Coble PA, Hayes AJ (1994). The Pittsburgh sleep diary. J Sleep Res.

[70] Barge-Schaapveld DQCM, Nicolson NA, Berkhof J, DeVries M (1999). Quality of life in depression: daily life determinants and variability. Psychiatry Res.

[71] Thewissen V, Bentall RP, Lecomte T, van Os J, Myin-Germeys I (2008). Fluctuations in self-esteem and paranoia in the context of daily life. J Abnorm Psychol.

[72] van Roekel E, Bennik EC, Bastiaansen JA, Verhagen M, Ormel J, Engels RCME, et al. Depressive symptoms and the experience of pleasure in daily life: An exploration of associations in early and late adolescence. J Abnorm Child Psychol. 2015;1–11. doi:10.1007/s10802-015-0090-z. 10.1007/s10802-015-0090-z PMC4893355 26496738

[73] Wichers M, Peeters F, Rutten BPF, Jacobs N, Derom C, Thiery E (2012). A time-lagged momentary assessment study on daily life physical activity and affect. Health Psychol Off J Div Health Psychol Am Psychol Assoc.

